# A Serological Survey of Selected Papua New Guinea Blood Donors for Hepatitis B and Related Co-Infections

**DOI:** 10.3390/tropicalmed5030108

**Published:** 2020-06-29

**Authors:** Francisca Varpit, Bruce Gummow

**Affiliations:** 1 Nonga General Hospital Laboratory, Kokopo 613, ENBP, Papua New Guinea; lakuadavina@gmail.com; 2 Discipline of Medical Laboratory Science, Division of Health Sciences, School of Medicine and Health Sciences, University of Papua New Guinea, PO O Box 5623, Boroko 111, NCD, Papua New Guinea; 3 Medical and Veterinary Sciences, College of Public Health, James Cook University, Townsville 4811, Queensland, Australia; 4 Australian Institute of Tropical Health and Medicine, James Cook University, Townsville 4811, Queensland, Australia; 5 Faculty of Veterinary Science, University of Pretoria, Pretoria 0002, South Africa

**Keywords:** Hepatitis B, human immunodeficiency virus, Syphilis, Hepatitis C, Co-infections, blood donors, Papua New Guinea

## Abstract

Hepatitis B virus (HBV) infection is a serious problem and earlier studies in Papua New Guinea have reported a high prevalence of hepatitis B virus infection. These studies were undertaken using insensitive tests and before an expanded immunization program. The current HBV status is therefore uncertain. A retrospective study to investigate the HBV status was carried out using blood donor data at Nonga General Hospital, East New Britain Province, Papua New Guinea, from January 2003 to December 2018. Additional data for Human Immunodeficiency Virus, syphilis and hepatitis C virus were also collected. Data were analysed using NCSS statistical software. The mean hepatitis B antigen (HBsAg) sero-prevalence was 21% for the period of study and showed a downward trend over the period of the study, which may reflect the effect of the extended immunization program. HBsAg prevalence in male donors (23%) was significantly higher than females (16%). Donors living in Pomio district had a significantly lower proportion of sero-positive HBsAg donors (7%) than Gazelle (22%), Kokopo (22%) and Rabaul (20%), which was attributed to this district’s geographical isolation. Ethnically, Pomios donors (8%) had significantly lower HBsAg prevalence than the Taulils, (29%), Bainings (21%) and Tolais (21%). Fifteen to nineteen year olds (23%) were the predominant age group affected, and vertical or perinatal transmission was probably the primary transmission route. Our findings call for greater awareness on the part of public policy makers and should be considered when planning future public health campaigns.

## 1. Introduction

Hepatitis B (HBV) infection is a serious problem both globally and nationally. Earlier studies in certain areas of Papua New Guinea (PNG) reported a high prevalence of Hepatitis B virus infection, especially in the highlands of PNG [1,2,3], the Sepik province [4] and in the Autonomous Island of Bougainville [5]. These studies were undertaken in the general population using insensitive tests and before an expanded immunization program. The HBsAg prevalence among blood donors in Papua New Guinea was reported in Port Moresby Blood transfusion services as 22.7% [6], and in the whole country in 1996 as 15% [7]. In about the same period, 24% of blood donors were reported positive for the HBsAg in Bougainville [8]. A study undertaken in 1998 that reviewed the HBsAg sero-positivity status in blood donors of Pacific countries stated the prevalence of HBV in PNG was 16.7%, second highest to the Solomon Islands (19.5%) [9]. Despite the high prevalence of HBV antibodies found in populations in Papua New Guinea [1,2,3,4,5,6,7,8,9], a study in 1987 looking at the aetiology of jaundice in a medical ward of one of the Hospitals in PNG failed to detect viral hepatitis in any of the clinical cases with jaundice [6]. This is in contrast to an earlier study published in 1976, which had examined 17 clinical cases, eight of which were strongly suggestive of viral hepatitis clinically [10]. Since the introduction of neonatal hepatitis B Immunoglobulin (hep B Birth Dose) immunization in 1989 and subsequent addition of the national three-dose infant hepatitis B (hep B3) vaccination program in 1992 [11], little work to establish the prevalence of HBV has been conducted in blood donors in Papua New Guinea. A recent comparative retrospective study in blood donors in Port Moresby General Hospital found a hepatitis B antigen (HBsAg) prevalence of 22.9% in blood donors in 2016 [12]. Co-infections were also indicated in the latter study at 7.3% (20/275) and were mostly seen in Family replacement donors. Co-infections were mainly with Human Immunodeficiency Virus (HIV) and syphilis [12], owing to shared means of transmission [13].

**Figure 1:**
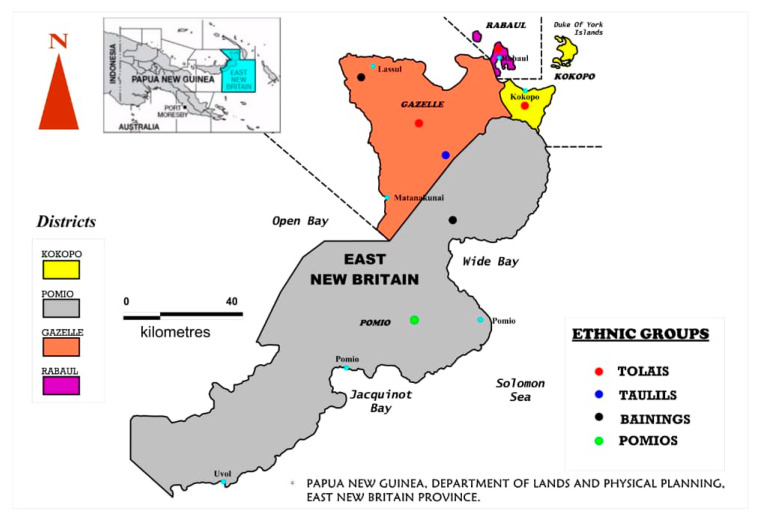
Map of East New Britain Province, Papua New Guinea; Map drawn by and with permission from; Ashley Lais (2020). Lands Department, East New Britain Province, Papua New Guinea.

East New Britain province (ENBP) is one of the five island provinces of PNG that lies between four and six degrees south of the equator (Figure 1). It has a total population of 327,355 people with a high literacy rate (86.6%) among the four island provinces and second in the country to the National Capital District (92.4%) [14]. It is also known as the fastest growing province in the country economically [15]. It has four districts inhabited by four distinct ethnic groups, namely the Tolais, Pomios, Taulils and the Bainings, which are all Melanesians (Figure 1). The Kokopo and the Rabaul districts are semi-urban areas and are inhabited predominantly by the Tolais, the Gazelle district is inhabited by the Tolais, the Taulils and the Bainings, while the Pomio district is inhabited predominantly by the Pomios. The current HBV status in ENBP is uncertain, and therefore this study was undertaken to establish the prevalence of HBV and related co-infections in blood donors to help with policy making in the province.

## 2. Materials and Methods

A retrospective observational cross-sectional study to investigate the HBV, HIV, hepatitis C virus (HCV) and Syphilis status of blood donors and related epidemiological risk factors in ENBP was carried out using blood donor data at Nonga General Hospital (NGH), ENBP, PNG from January 2003 to December 2018. Mandatory pre-screening for health and social status are done on all prospective blood donors presenting for donation at the blood Transfusion Service. This is in concordance with the PNG National Health Department Blood Policy, in partnership with the World Health Organization [16].

Epidemiological variables included in the data analysis were age, gender, district of origin, ethnicity, infection status and diagnostic tests. Ethnicity was based on the village that was recorded in the database for that patients place of residence, with the assumption that people from a particular village would all be of the same ethnicity, since tribal clustering is still relevant to PNG. Infectious status was determined in the hospital as part of their diagnostic service using commercially available kits and relevant demographic data were recorded at the time. The following kits were used by the hospital for diagnostic purposes (note that this study only looked at the results of the tests and the authors were not involved in the diagnostic process). The Gelatin Particle Agglutination assay using Serodia ^®^-HBs PA (specificity 100%; sensitivity 99.0%. Fujiebio Inc., Tokyo, Japan) was used to detect HBsAg before 2010. This test had a reported minimum detection limit of 0.5 ng/mL HBsAg. After 2010, immunochromatographic assays were used for HBsAg detection, which included DetermineTM HBsAg (sensitivity 100%; specificity >98%. Abbott Laboratories, Dainabot Co. Ltd., Tokyo, Japan). Alternatively, the Hepa S-Ag test kit using reverse passive hemagglutination (specificity 100%; sensitivity 99%. Green Cross Medical Science, Yongin, South Korea) or the Chembio Diagnostic Systems kit (specificity 100.0%; sensitivity 99.0%. Medford USA) was used, depending on availability. Test kits used were in keeping with World Health Organization (WHO) recommended assay required for EIAs (sensitivity 100%; specificity ≥98%) and RDTs (sensitivity ≥99%; specificity ≥98%) [17]. WHO recommended limit of detection for both EIAs and RDTs is ≤0.13 IU/mL.

The HIV antibody rapid screening kit, Determine™ HIV-1/2 (specificity 99.87%; sensitivity 100%, Abbott Laboratory, IL, USA) was used together with the Immuno Comb^®^ II HIV 1 and 2 kit (specificity 99.4%; sensitivity 100%. Bispot kit PBS Organics and Israel 2005,) to screen for HIV. This was followed in series by the SERODIA^®^-HIV-1/2 test kit for HIV, which is an in vitro diagnostic test kit for the detection of antibodies to HIV type1 and/or type 2 (HIV-1/HIV-2) in human serum/plasma (specificity 100%; sensitivity 100%. Fujirebio Inc., Japan).

Two tests were used for the screening of Syphilis; the Syphilis TPHA liquid Hemagglutination test for qualitative and quantitative detection of *T. pallidum* antibodies in serum specimens (sensitivity 98.5%; specificity 100%. Hexagon, Germany) or the Syphilis RPR Rapid test for qualitative and semi--quantitative detection of antibodies, associated with syphilis, in serum or plasma (specificity and sensitivity not given by manufacturer. HUMAN Gesellschaft für Biochemica und Diagnostica mbH Max-Planck-Ring 21 65205 Wiesbaden, Germany). In addition, the SD BIOLINE Syphilis 3.0 test was used to test for Syphilis, which is a solid-phase immunochromatographic assay for the qualitative detection of antibodies of all isotypes (IgG, IgM, IgA) against Treponema pallidum (TP) (sensitivity 99.3%; specificity 99.5%. Standard Diagnostic, Korea,). The SD BIOLINE HCV (02FK10, 02FK16, 02FK17) chromatographic testing kit was used to test for the HCV in 2018 blood donors (sensitivity 100%; specificity 100%. SD Standard Diagnostics, Inc., Korea,). This test was introduced to all blood bank centres in PNG in 2018, and therefore only HCV test results for 2018 are reported for our study. Other tests used for the diagnosis of active hep B and C infections were not performed in this setting because of financial constraints. Positive donors for HBsAg were excluded from repeat donation, however, donors were not always informed of their results unless they came back for their results or came back for a second donation. Additionally, all tests positive for HBsAg were not repeated.

Apparent prevalence (AP) and its corresponding 95% confidence interval (CI) were calculated using proportions of donors at NGH, testing positive on one or more of the diagnostic tests described above. The Chi-Square test was used to look for significant associations between the proportion of HBsAg seropositive donors and demographic variables. The level of significance was set at *p* ≤ 0.05%. Odds Ratios were calculated to determine the strength of association between the proportion of seropositive HBsAg donors and demographic variables.

For the multivariable logistic regression models, survey data were entered into Microsoft Excel (2007) and the computer software program NCSS (NCSS Inc., Kaysville, UT, USA) for analysis. The associations between seropositive HBsAg donors and various risk factors were analysed using Chi-square tests (i.e., univariate analysis). Risk factors with a *p*-value < 0.20 on univariate analysis were selected to be included in the multivariable logistic regression models [18]. A hierarchical stepwise forward elimination process with switching of variables based on log likelihood values was used to determine the best fitting logistic regression model. NCSS has a built-in algorithm that does the selection process and several iterations were run before the final model was selected, which contained the best fitting log likelihood value and only the variables with a *p*-value < 0.05 on the Wald test [19,20]. Due to collinearity between the District and Ethnic groups, two separate models were run with the inclusion of only one of these variables in each model.

Ethical Clearance was granted by the Nonga General Hospital Management on 6 February 2017 and the University of Papua New Guinea School of Medicine & Health Sciences Research Ethics Committee (UPNG SMHS REC) approved the research on 10 October 2016. All records collected were de-identified and only data required for analysing trends was collected.

## 3. Results

A total of 24,478 donor records from 2003–2018 were analysed. Of these, 41.4% (n = 10,128) were excluded because of missing variables of interest. Of the remaining 58.6% (n = 14350), 67.5% (n = 9686) were males and the rest were females (32.5%, n = 4664). The average number of donors per year and standard deviation over the period studied was 897 ± 497 and ranged from 129 in 2017 to 1832 in 2003 (Appendix A).

The mean ages and standard deviations for males and females were 31 ± 11.6 and 31.8 ± 11.3 years, respectively. Though there was no statistical difference observed between the mean age of males and females, the age range was 74 and 58 years, respectively. In both genders, 20-year olds donated more frequently than the other ages. More males continued to donate annually throughout the study period.

**Figure 2:**
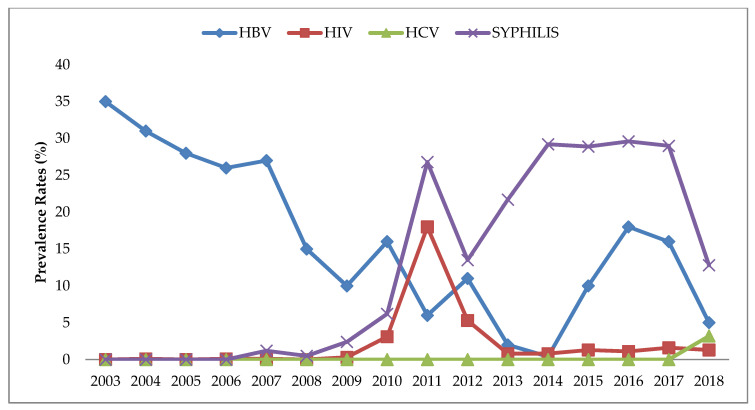
The trend of single infection by hepatitis B virus (HBV), human immunodeficiency virus (HIV), Syphilis and hepatitis C virus (HCV) from 2003–2018, based on calculated estimated prevalence rates.

**Figure 3:**
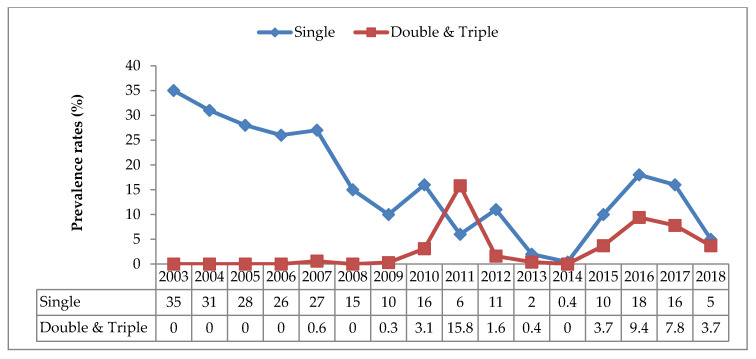
Comparison of trends of HBV single and co-infection prevalence rates in blood donors from 2003–2018.

The prevalence of sero-positive HBsAg donors with a single infection for the period of the study (2003–2018) was found to be 19% (95% CI 18.9–20.2%). Trends showed a decline from 35% (95% CI 33.0–37.4) in 2003 to 0.4% (95% CI −0.1–0.9) in 2014 before rising again in 2015 and 2016. Single infections with syphilis and HIV showed increasing trends to 2011. After a decline in 2012, syphilis infections increased in 2013 and 2014, and remained constant in the next 3 years, and declined in 2018 (Figure 2, Appendix A, Table A1). Because of common modes of transmission, an increase in positive cases of HIV and Syphilis infection in 2015 and 2016 may have also impacted on the increase in HBsAg prevalence during the same time period. The prevalence of double and triple infections was found to be 2% (95% CI 2.0–2.2%). The prevalence of sero-positive HBsAg donors with a double or triple infection increased from 0.6% (95% CI 0.23–0.99%) in 2007 to 15.8% (95% CI 13.3–18.2) in 2011 before declining in 2012 from 1.6% (95% CI 0.5–2.7%) to 0% in 2014, then rising again in 2016 (Figure 3).

**Figure 4:**
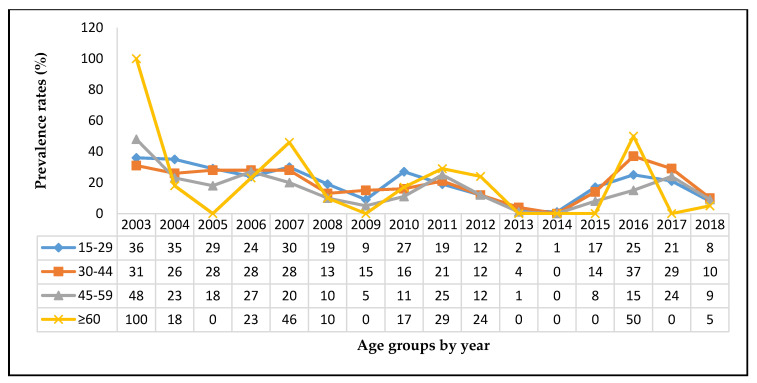
Annual prevalence rates of HBV in blood donors from 2003–2018 by age group.

The annual distribution of HBsAg infections for each age group shows declining hep B virus infection with increasing years (Figure 4, Appendix A Table A2). In 2003, the HBsAg prevalence in 15–29-year age group was 36% (95% CI 33–38) and declined to 9% (95% CI 5–12) in 2009. An increase was again observed in 2010 (27%; 95% CI 22–32); which again declined to 1% (95% CI −0.3–2) in 2014. In 2015, a rise in prevalence was again observed (17%; 95%CI 10–24); which continued to increase in 2016 (25%; 95% CI 19–31) then started to decline again in 2017 (25%, 95% CI 11–32) and 8% (95% CI 5–19) in 2018. The same pattern was observed in other age groups. This may have reflected the introduction and fluctuating trends in the neonatal hep B Immunoglobulin immunization coverage in PNG [20].

**Figure 5:**
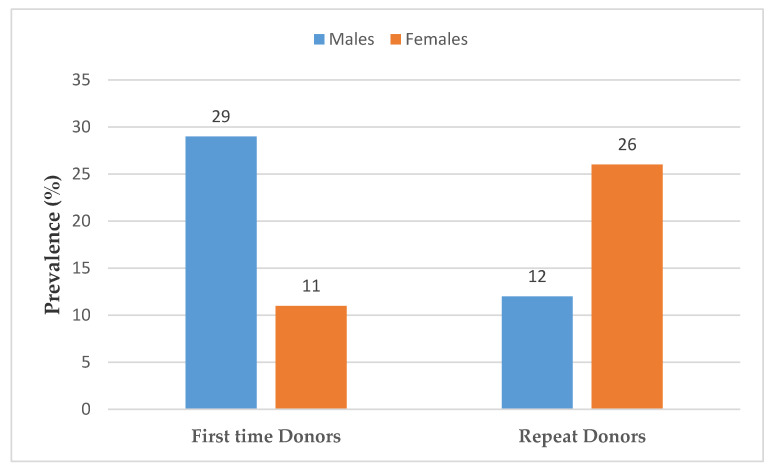
Proportion of HBsAg positive first time and repeat blood donors between 2003–2018.

The prevalence in sero-positive HBsAg first time donors (23%; 95% CI 21.6–23.4) was significantly higher than repeat donors (16%; 95% CI 14.8–16.6 (Table 1). This appears to be linked to the large number of male donors as when stratified by gender, the proportion of HBsAg sero-positive first-time male donors (29; 95% CI 27.7–30.2) was higher than repeat male (12; 95% CI 10.6–12.5) donors, yet the proportion of HBsAg positive repeat female donors (26; 95% CI 24.3–28.4) were) was higher than first time female donors (11; 95% CI 9.7–12.0) (Table 2, Figure 5). The significantly higher HBsAg seroprevalence observed in first time donors could be due to the fact that repeat donors positive for the hep B virus are almost always exempted from donation.

The highest proportion of sero-positive HBsAg donors was aged 15–29 years (23%, 95% CI 22.5–23.4). Donors living in Pomio district had a significantly lower proportion of sero-positive HBsAg donors (7%; 95% CI 5.2–9.3) than Gazelle (22%; 95% CI 22.0–22.9), Kokopo (22%; 95% CI 21.2–22.8) and Rabaul (20%; 95% CI 15.3–20.7). Ethnically, the Pomio people had a significantly lower proportion of sero-positive HBsAg donors (8%; 95 CI 6.2–10.6) than the Bainings, Taulis and Tolais (Table 1) but this could be related to district, as ethnicity tended to be clustered according to the district they lived in. Co-infections of sero-positive HBsAg donors with syphilis were more common than co-infections with HIV, HCV and triple infections (Table 1). When stratified by gender, the proportion of sero-positive HBsAg male donors (21%; 95% CI 20.5–21.3) was significantly higher than female donors (16%; 95% CI 16.0–17.3), with the highest prevalence in the Taulils males (35%; CI 30.1–41.1) (Table 2). The prevalence of sero-positive HBsAg donors was lowest in both Pomios men and women (Table 2).

Two logistic regression models were done to identify risk factors in the districts and among ethnic groups since district and ethnic groups showed collinearity. Model one (Table 3), which includes district, uses Gazelle, which has the highest prevalence of HBsAg, as a reference and shows that Rabaul (OR = 0.78) and Pomio (OR = 0.27) both had lower odds of having donors positive to HBsAg than Gazelle. No difference in HBsAg prevalence could be shown between Kokopo and Gazelle districts. In this model, there is no significant association between HBsAg-positive donors and those positive for HCV or HBsAg (*p* = 0.06). There was a strong association between HBsAg positive donors and donors positive for HIV (OR = 4.7). Age groups 30–44 years (OR = 0.8) and 45–59 years (OR = 0.6) were less likely to be HBsAg positive than the 15–29-year age group. There was no difference in risk between 15–29 and ≥60 years old, but this may be due to the small number of ≥60-year-olds in the survey. Males were more likely to be HBsAg positive than females (OR = 1.3). The final model 1 was therefore: HBsAg = 1 = 0.00 − 0.03*(DISTRICT = “KOKOPO”) − 1.30*(DISTRICT = “POMIO”) − 0.25*(DISTRICT = “RABAUL”) + 1.55*(HIV = 1) − 0.23*(Age_2 = 2) −0.59*(Age_2 = 3) − 0.33*(Age_2 = 4) + 0.27*(Gender = “M”). The model was found to correctly classify HIV seropositive donors 51.7% of the time.

Model 2 (Table 4) that includes ethnicity shows that previous donors are less likely to be HBsAg positive (OR = 0.8). Pomio donors are less likely to be HBsAg positive than the Bainings (OR = 0.57) and no difference in risk can be shown between the Taulils and the Bainings or between Tolais and Bainings. Unlike, Model 1, donors positive for HCV were shown in this model to have less chance of being HBsAg positive (OR = 0.39), which means district was probably confounding this relationship. HCV exposure may therefore be associated with ethnicity. Male donors were confirmed to be more at risk of having HBsAg sero-positivity than females (OR1.3). The model confirmed that old donors were less likely to be HBsAg positive (OR = 0.84) than new donors. The final model 2 was, therefore, HBsAg = 1 = −0.02 + 0.29*(Gender = “M”) + 1.58*(HIV = 1) − 0.18*(OLD_NEW = “OLD”) − 0.21*(Age_2 = 2) − 0.57*(Age_2 = 3) − 0.28*(Age_2 = 4) − 0.56*(ETHNICITY = “POMIO”) + 0.21*(ETHNICITY = “TAULIL”) + 0.00*(ETHNICITY = “TOLAI”) − 0.94*(HCV = 1). The model was found to correctly classify HIV seropositive donors 55.6% of the time.

## 4. Discussion

The mean calculated HBsAg prevalence in this study is 23% (95% CI 23.0–24.0) and is similar to other studies found in the region before an expanded programme for immunization was introduced [5,21,22,23,24]; regional prevalence’s varied from 17.9% in Fiji [22], 32% in British Solomon Islands [21] and Kiribati [23], 42% in Tahiti [5] and 88% in Tonga [24].

Our study demonstrated a downward trend in HBsAg sero-prevalence from 2003–2018 (Figure 2, Table 1). This is in accordance with studies done in the Solomon Islands, which demonstrated a decline in HBsAg prevalence from 32% in 1977 [21] to 25.1% in 1999 [25] to 21.5% in 2007 [26]. Similarly, Fiji has seen a decline in prevalence from 17.9% in 1982 [22] to 0% in children, 5.6% in adolescents and 3.2% in adults in 2009 [27]. The decline in donor HBsAg prevalence seen in our study, from a high of 35% in 2003 to 5% in 2018 in ENB province, can probably be attributed to the expanded program for immunization in the province and increased awareness of the disease within communities as a result of these campaigns. The fluctuating decline in donor HBsAg prevalence infections may have been indirectly affected by fluctuating hepatitis B immunization coverage rates, which varied from 42% in 2001 to 61% in 2002, 68% in 2003 and a decline to 61% in 2004. In 2005, coverage increased again to 67% and 75% in 2006. In 2007 and 2008, a decline in coverage was again recorded from 66–65%, respectively [28]. Furthermore, the estimation of hep B Birth dose (hep BB) and hep B3 (triple dose) immunization coverage rates in PNG showed declining rates from 26% in 2009 to 25% in 2018, and 66% in 2009 to 61% in 2018, respectively [29].

### 4.1. HBV Infection among the Districts and Ethnic Groups of This Study

The Gazelle, Kokopo and Rabaul districts had the highest prevalences of HBsAg at 22%, 22%, and 20% respectively, which were twice the Pomio district (Table 1). The Rabaul and the Kokopo districts are both semi-urban areas, while the Pomio and Gazelle districts are mostly rural. Though both the Pomio and the Gazelle districts are rural settings, HBsAg seropositivity in the Pomio district was lower than the Gazelle district (Table 2). In fact, Pomio district has lower odds (OR = 0.3) of having HBsAg infection than Gazelle (Table 3). A possible factor that may explain this difference in HBsAg prevalence between Gazelle and Pomio districts could, therefore, be the difference in the ethnicity of donors in the two areas, with most donors in Gazelle being predominantly Tolais and Bainings and some Taulils, while the Pomio district is populated almost exclusively by Pomios. In other studies [30,31,32], familial clustering is a common risk factor among rural inhabitants, or intra-familial transmission [33].

Inhabitants of the two semi-urban districts of Rabaul and Kokopo are either located within the vicinity of a hospital or have easy access to a hospital and blood donation services due to good road networks, unlike most areas of the Gazelle and Pomio districts. It is not clear from our results, however, if this had an influence on exposure rates. Although both Rabaul and Kokopo districts are semi-urban, Rabaul has slightly lower odds of having donors positive for HBsAg than Gazelle, but no difference in risk could be shown between Kokopo and Gazelle. The people in Rabaul and Kokopo districts are predominantly Tolais as is the majority of Gazelle, which could explain the relatively small difference in prevalence of HBsAg between these districts rather than socioeconomic factors. The fact that Bainings were clustered together with Taulils and Tolais in Gazelle and were not living in other districts could explain why no difference in HBsAg prevalence could be shown between these ethnic groups. It is difficult to explain variations between these ethnic groups, but it could mean that accessibility to basic health services is not a key factor. Alternatively, isolation of the Pomios, who are mostly accessible only via air and water transport, may have mitigated against spread to this population. Lifestyle was seen to be a factor in two rural populations of New Caledonia [34], yet a study in Fiji showed high prevalence that appeared not to have been affected by location and lifestyle [21]. A study in Tonga showed increased new cases of HBV infections in rural dwellers compared to urban and attributed this to a lifestyle in rural settings that predisposed them to increased infection [35]. While this may hold true for the Taulils, Bainings and Tolais of the Gazelle district, it is in contrast to the Pomios in this current study, who live mostly in rural areas and yet have the lowest HBsAg prevalence.

The reason why HBV is so prevalent in Tolais is complex but one reason could be that when the Tolais migrated from nearby New Island Province (NIP) to settle in ENBP, they could have carried the virus along with them and introduced it to those who were already there. There is a possibility, therefore, that genetic predisposition could be playing a role amongst the ethnic groups in this study as well.

The overall literacy rate in the province is high [14] and, in other studies, high literacy rates have been found to be associated with decreasing HBV infection among rural populations [36,37]. This is in contrast to this study where the Pomio district has a lower literacy rate as compared to the rest of the districts in the province and yet has the lowest HBsAg prevalence and odds of being HBsAg positive, while the Gazelle district has a high literacy rate [38] and yet has a significantly higher HBsAg prevalence than Pomio. 

The logistic regression models generated in this study only had a correct classification rate of 51 to 57%, indicating that numerous other risk factors not included in the models were likely playing a role. Other factors, such as poor knowledge and attitude about HBV infection, the importance of vaccination against it, parents being older, and families with low monthly income have all been identified as having an association with higher HBV prevalence rates [37,39,40].

### 4.2. HBV Infection among Each Gender and Sub-Age Groups

The disease is more prevalent in males than in females in our study (Table 2). Males are also more likely to be positive for HBsAg than females (OR = 1.3). This is in contrast to Nkrumak et al. [41], whose study demonstrated higher HBsAg prevalence in females than males (21.4%, 95%CI 11.6–34.4 vs. 13.2%, 95%CI 10.8–15.9). More males than females were presenting for blood donation in this study, which is consistent with other similar studies [42,43], and this may be confounding the prevalence of HBsAg in males. However, the higher prevalence of positive males could also be due to increased male susceptibility to infection [21] and a gender tendency to chronicity [4]. In this study, HBsAg infection is lower in the Pomio district among both genders. One possible reason that Pomios males have less infection could be due to male circumcision practiced in this ethnic group. This practice reduces the risk of transmission sexually transmitted diseases, as seen in another study [42]. A reason why females are less at risk, as given by Mazzur and Jones [21], is that females do not venture out of the home to socialize as often as males.

HBV exposure was seen in our results to be highest among adolescents and young adults in both genders (Table 1). This is similar to a study done in Bougainville [5] but in contrast to an earlier long-term study in the Sepik district, where HBV infections increased with age in all populations studied [3]. Though both Hawkes et al. and Wong et al. [4,5] also employed other markers of past infections such as the anti-HBc, which increased their detection rates, the general scenario was that, for both genders, evidence of HBV infection increased with age, although the positivity rate of increase varied. This indicated vertical and horizontal acquisition from an early age [21,34,43,44,45,46]. In this study, high levels of HBsAg were seen among teenagers and young adults, the 15–29-year-olds. The highest prevalence of HBV infection was observed in 2003 among 15–29-year-olds. This may be because donors aged 16–29 years during this time would not have been vaccinated against the virus, as the vaccine was first introduced into the country in 1989. This suggests that acquisition was mostly vertical or perinatal. This is further supported by the fact that IV drug use is rare in these communities.

It could not be concluded in this study if the HBsAg positive donors were chronic carriers as repeat donors positive for HBsAg were not followed up.

### 4.3. HBV Co- and Triple Infection with Syphilis, HIV and HCV

According to the World Health Organization [47], about 2.7 million (1%) or 7.4% of people with HBV are HBV/HIV-co-infected. Our study found that only 0.7% of HBV positive donors co-infected with HIV. However, donors infected with HIV were more likely to be positive for HBsAg (OR = 4.8), no significant association between donors positive for HCV and HBsAg was seen. This means that donors positive for HCV are less likely to be infected with HBV (OR = 0.39).

Many studies have demonstrated continuous occurrence of HBsAg co-infections with HIV apart from syphilis, especially among asymptomatic MSM [48,49,50,51,52,53,54,55,56], sex workers [49,50], high-risk sexual behaviour individuals [48,54] and among the immuno-compromised population [54,55,56,57]. Furthermore, it has been found that the risk of developing HBV chronicity is 5–6 times higher in individuals positive for HIV than in those negative for it [52]. HIV infection has also been indicated to be increasing in ENBP [58] and in the country as a whole at an exponential rate [59] and is most common among the young adults of 20–29 years of age [60]. In our study and others [21,43], this is also the sub-age group in which HBsAg is significantly prevalent, which is a cause for concern. Although HBV/HIV co-infection seems to be relatively low in this study as compared to others [61,62], it is imperative for blood banks in PNG to take note that escape mutants in donors can escape being detected using conventional assays, posing a greater risk to recipients.

The prevalence of HBsAg/Syphilis and HBsAg/HIV co-infections in this study was significantly higher than with HCV and triple infections. More males were co-infected with syphilis than females, while females were more commonly co-infected with HIV than with syphilis and HCV. Triple infection was more common among males than females (Table 2). This is in contrast to Butsashvili et al. [53], who did not find any association between being positive for HBsAg and having antibodies against *Treponema pallidum* among blood donors. Triple infections with HIV and syphilis have also been observed in other studies in asymptomatic high-risk individuals such as MSM and sex workers and is attributed to people not knowing if they are infected [54,55]. A lack of knowledge about having been infected has been found to result in 3.5 times higher risk than those who know their serological status [63]. The feedback of results to patients is therefore important in combating these sexually transmitted diseases.

## 5. Conclusions

Hepatitis B virus was still prevalent in the overall population in ENBP in 2018 despite existing control measures, which call for greater awareness on the part of public policy makers in the province, since it is possible that low levels of escape mutants will remain undetectable in blood donor blood and may pose a threat to the recipients as has been demonstrated in other studies [59,60]. Transmission of HBV in this study is likely to be perinatal or vertical, as demonstrated by annual high levels of HBsAg prevalence in young adults (15–29 years) from 2003–2018 and supported by the fluctuating hep B immunization coverage over the years [28]. Male donors are at a greater risk of being infected with HBV than females, which may be attributed to lifestyle. The Pomios people living in the Pomio district had a significantly lower prevalence of HBV than Kokopo, Rabaul and Gazelle districts or the Tolais, Taulils and Bainings people, which was attributed to their remoteness and isolation from other ethnic groups. Co-infections with HIV in females and syphilis in males is of concern and efforts to make donors aware of their infective state should be a priority at hospitals testing for these diseases.

These findings should be considered when planning public health campaigns and high-risk groups need to be targeted for intervention and existing control measure reviewed.

## 6. Limitations

Syphilis testing only started in 2007 and therefore an accurate conclusion on reactive syphilis cases could not be drawn from this study. Within the period that syphilis was being tested for, some analysis was not done periodically due to reasons such as a shortage of reagents or inconsistencies on the part of the laboratory personnel responsible for the analysis. A total of 32 (0.35%) of the total population studied had an initial reactive test for HIV and had to undergo confirmatory testing but were never confirmed, and therefore the percentage of positive cases of HIV in blood donors in this study could have been higher. Risk factors for the possible ethnicity of blood donors were identified based on the location (villages) in the districts where their blood was drawn during blood collection and therefore, the likelihood of misidentifying someone as belonging to that ethnic group is possible but would constitute a small portion of the sample due to clustering of people along tribal lines in PNG. Another limitation of this study is the inability to trace repeat donors, and therefore it may have been possible that some donors had donated more than once but were treated as new donors. Additionally, seroconversions were not identified and therefore the same people may have been counted more than once.

Although the specificity and sensitivity of assay kits employed were indicated to be at WHO-approved levels, limits of detection were not indicated, and therefore this could have impacted on the results obtained over the years. Consistency in the use of a single assay needs to be improved, and existing criteria for follow-up of donors positive for HBsAg need to be reviewed.

## Figures and Tables

**Table 1:** Mean prevalence rates of HBV infections by age groups, donor frequency, districts, ethnicity and co-infections in East New Britain province between 2003–2018.

Demographic	Proportion Positive	Prevalence % (95% CI)	x²	*p*-Value
Total HBsAg Infection	3102/14350	21 (21.0–22.0)		
Single infection	2804/14350	19 (19.0–19.8)		
Age group				
15–29	1669/7272	23 (22.5–23.4)		
30–44	843/4809	17 (17.0–18.1)	130.41	˂0.001
45–59	270/2100	13 (12.0–13.6)		
≥60	22/169	6 (4.6–8.4)		
Donor frequency				
Repeat donor	986/6269	16 (14.8–16.6)	102.89	˂0.001
First time donor	1818/8081	23 (21.6–23.4)		
District				
Kokopo	622/2826	22 (21.2–22.8)		
Gazelle	1775/7904	22 (22.0–22.9)	27.38	˂0.001
Pomio	11/152	7 (5.2–9.3)		
Rabaul	694/3468	20 (19.3–20.7)		
Ethnicity				
Bainings	99/360	21 (19.7–23.5)		
Pomios	13/142	8 (6.2–10.6)	19.51	˂0.001
Taulils	30/73	29 (24.7–33.5)		
Tolais	2960/10673	21 (21.0–22.1)		
Co & Triple infection				
HBsAg/SYPHILIS	120	0.8 (0.6–1.0)		
HBsAg/HIV	101	0.7 (0.5–0.9)	38.21	˂0.001
HBsAg/HCV	65	0.5 (0.3–0.6)		
Triple Infection	12	0.1 (0.0–0.2)		
Total	298/14350	2 (2.0–2.2)		

**Table 2:** Mean prevalence rates of HBsAg by gender for single and co-infections among districts, ethnic groups and age groups in East New Britain province between 2003–2018.

	Males			Females		
Demographic	Proportion	Prevalence %	Proportion	Prevalence %	*X*²	*p*-Value
	Positive	95 CI%	Positive	95% CI		
Gender	2236/9686	23 (22.6–23.5)	866/4664	18 (18.0–19.1)	39.91	˂0.001
Single HBsAg Infection	2023/9686	21 (20.5–21.3)	781/4664	16 (16.0–17.3)		
Donor Frequency						
Repeat Donors	517/4487	12 (10.6–12.5)	469/1782	26 (24.3–28.4)	714.58	˂0.001
First Time Donors	1506/5199	29 (27.7–30.2)	312/2882	11 (9.7–12.0)		
District						
Kokopo	428/1924	22 (21.3–23.2)	194/902	21 (20.1–22.9)		
Gazelle	1270/5255	24 (23.6–24.8)	505/2649	19 (18.3–19.8)		
Pomio	8/101	8 (5.3–10.6)	3/51	5 (2.7–9.1)	45.99	˂0.001
Rabaul	530/2406	22 (21.2–22.9)	164/1062	15 (14.4–16.5)		
Ethnicity						
Bainings	70/294	23 (21.4–26.3)	29/165	17 (14.7–20.5)		
Pomios	9/103	8 (6.0–11.5)	4/52	7 (4.1–11.3)	26.11	0.002
Taulils	26/73	35 (30.1–41.1)	4/30	13 (7.2–19.4)		
Tolais	2131/9216	23 (22.7–23.6)	829/4417	18 (18.0–19.4)		
Age Group						
15–29	1306/5120	25 (24.9–26.1)	483/2152	22 (21.6–23.3)		
30–44	663/3054	21 (21–22.4)	281/1755	16 (15.2–16.9)	183.98	˂0.001
45–59	236/1366	17(16.3–18.3)	99/734	13 (12.3–14.7)		
≥60	31/146	21 (17.9–24.6)	23-Mar	13 (6.1–19.9)		
Double and Triple HBsAg Infections						
HBsAg/Syphilis	100	1.0 (0.8–1.2)	20	0.4 (0.2–0.6))		
HBsAg/HIV	67	0.7 (0.5–0.9)	34	1.0 (0.5–1.0)		
HBsAg/HCV	36	0.4 (0.3–0.5)	29	0.6 (0.4–0.9))	149.1	˂0.001
Triple Infection	10	0.1 (0.0–0.2)	2	0.0 (0.0–0.1)		
Total	213/9686	2 (2.0–2.4)	85/4664	2 (1.6–2.0)		

**Table 3:** Multivariable logistic regression model 1 (excluding ethnicity) for risk factors associated with HBsAg positive blood donors in east New Britain province (ENBP), 2003–2018.

Independent Variable	Regression Coefficient	SE	Wald Z-Value	Wald *p*-Value	Odds Ratio	Lower 95% CI	Upper 95% CI
B0: Intercept	0.00	0.04	−0.02	0.99	0.99	0.92	1.08
DISTRICT = “KOKOPO”	−0.03	0.05	−0.50	0.62	0.97	0.88	1.08
DISTRICT = “POMIO”	−1.30	0.32	−4.10	<0.01	0.27	0.15	0.51
DISTRICT = “RABAUL”	−0.25	0.05	−4.736	<0.01	0.78	0.70	0.86
HIV = 1	1.55	0.13	12.353	<0.01	4.71	3.68	6.02
Age = 15–29 years vs. 30–34 years	−0.23	0.05	−4.873	<0.01	0.80	0.73	0.87
Age = 15–29 years vs. 25–59 years	−0.59	0.07	−8.770	<0.01	0.55	0.48	0.63
Age = 15–29 years vs. ≥60 years	−0.33	0.20	−1.683	0.09	0.72	0.49	1.06
Gender = ”M”	0.27	0.05	5.866	<0.01	1.31	1.19	1.43

**Table 4:** Multivariable logistic regression model 2 (excluding district) for risk factors associated with HBsAg positive blood donors in ENBP, 2003–2018.

Independent Variable	Regression Coefficient	Standard Error	Wald Z-Value	Wald Prob Level	Odds Ratio	Lower 95% C	Upper 95% C
B0: Intercept	−0.02	0.12	−0.12	0.90	0.99	0.78	1.25
Gender = “M”	0.29	0.05	6.27	<0.01	1.33	1.22	1.45
HIV = 1	1.58	0.13	12.57	<0.01	4.84	3.79	6.19
OLD_NEW = “OLD”	−0.18	0.04	−4.16	<0.01	0.84	0.77	0.91
Age = 15–29 years vs. 30–34 years	−0.21	0.05	−4.59	<0.01	0.81	0.74	0.89
Age = 15–29 years vs. 25–59 years	−0.57	0.07	−8.33	<0.01	0.57	0.50	0.65
Age = 15–29 years vs. ≥60 years	−0.28	0.20	−1.41	0.16	0.76	0.51	1.11
ETHNICITY = “POMIO”	−0.56	0.27	−2.03	0.04	0.57	0.33	0.98
ETHNICITY = “TAULIL”	0.21	0.25	0.86	0.39	1.24	0.76	2.01
ETHNICITY = “TOLAI”	0.00	0.12	0.03	0.98	1.00	0.80	1.26
HCV = 1	−0.94	0.48	−1.97	0.05	0.39	0.15	0.99

## Appendix A

**Table A1:** The annual prevalence rates of single infection for HBsAg, HIV, Syphilis and HCV in blood donors in East New Britain province from 2003–2018.

Year	HBsAg Single Infection		HIV Single Infection	
	n =	Prevalence	n =	Prevalence
		(95%CI)		(95% CI)
2003	645/1832	35 (34.11–36.30)	0/1832	0
2004	439/1414	31 (29.84–32.26)	2/1414	0.1 (0–0.3)
2005	200/712	28 (26.44–29.74)	0/712	0
2006	378/1462	26 (24.73–26.98)	1/1462	0.1 (0–0.2)
2007	451/1641	27 (27.00–29.18)	2/1640	0.1 (0–0.3)
2008	145/952	15(14.09–16.38)	0/952	0
2009	68/687	10 (9.33–11.63)	2/685	0.3 (0–0.8)
2010	144/882	16 (17.97–20.58)	27/867	3.1 (2.0–4.3)
2011	52/880	6 (20.34–23.07)	154/855	18 (15.4–20.6)
2012	87/820	11 (11.07–13.32)	41/781	5.3 (3.7–6.8)
2013	10/515	2 (1.68–2.98)	4/510	0.8 (0.02–1.6)
2014	2/531	0.4 (0.12–0.64)	4/528	0.8 (0–1.5)
2015	24/242	10 (11.47–15.80)	3/240	1.3 (0–2.7)
2016	78/445	18 (24.90–29.03)	5/442	1.1 (0.2–2.1)
2017	20/129	16 (19.60–26.91)	2/129	1.6 (0–3.7)
2018	60/1206	5 (4.44–5.68)	25/1888	1.3 (0.8–1.8)
Total	2804/14350	19.21–19.87	272/14237	1.9 (1.7–2.1)
**Year**	**Syphilis Single**	**Infection**	**HCV Single Infection**	
2003	0/1832	Not done	0/1832	0
2004	0/1414	Not done	0/1414	0
2005	0/712	Not done	0/712	0
2006	0/1462	Not done	0/1462	0
2007	20/1633	1.2 (0.7–1.8)	0/1641	0
2008	5/947	0.5 (0.1–1.0)	0/952	0
2009	16/674	2.4 (1.2–3.5)	0/687	0
2010	53/849	6.2 (4.6–7.9)	0/882	0
2011	217/810	26.8 (23.7–29.8)	0/880	0
2012	99/732	13.5 (11.1–16.0)	0/820	0
2013	92/425	21.7 (17.7–25.6)	0/515	0
2014	120/411	29.2 (24.8–33.6)	0/531	0
2015	56/194	28.9 (22.5–35.2)	0/242	0
2016	111/375	29.6 (25.0–34.2)	0/445	0
2017	31/107	29 (20.4–37.6)	0/129	0
2018	133/1037	12.8 (10.8–14.9)	37/1169	3.2 (2.58–3.56)
Total	953/13614	7.0 (6.6–7.4)	37/14313	0.3 (0.22–0.30)

**Table A2:** The annual distribution of single and co-infections of hepatitis B virus categorized by age groups in East New Britain province from 2003–2018.

Age Group (Years)	15–29	Prevalence	30–44	Prevalence	45–59	Prevalence	≥60	Prevalence	Annual	Prevalence
Year		95% CI		95% CI		95% CI		95% CI	Total	95% CI
2003	521/1460	36 (33–38)	98/320	31 (26–36)	24/50	48 (34–62)	2/2	100 (100–100)	649/1832	35 (33–38)
2004	284/804	35 (32–39)	125/477	26 (22–30)	28/122	23 (16–30)	2/11	18 (−5–41)	439/1414	31 (28–33)
2005	137/471	29 (25–33)	57/207	28 (22–34)	6′/33	18 (5–31)	0/1	0 (0–0)	200/712	28 (25–31)
2006	157/660	24 (21–27)	169/609	28 (22–34)	49/180	27 (21–34)	3/13	23 (0.2–46)	378/1462	26 (24–28)
2007	247/833	30 (27–33)	159/577	28 (24–31)	41/205	20 (15–26)	12/26	46 (27–65)	459/1641	28 (26–30)
2008	78/405	19 (15–23)	52/392	13 (10–17)	14/145	10 (5–15)	1/10	10 (-9–29)	145/952	15 (13–18)
2009	25/292	9 (5–12)	39/266	15 (10–19)	6/126	5 (1–9)	0/3	0 (0–0)	70/687	10 (8–13)
2010	91/339	27 (22–32)	61/385	16 (12–20)	16/146	11 (6–16)	2/12	17 (−4–38)	170/882	19 (17–22)
2011	58/300	19 (15–24)	63/305	21 (16–25)	63/251	25 (20–31)	7/24	29 (11–47)	191/880	22 (19–24)
2012	34/290	12 (8–15)	35/289	12 (8–16)	25/216	12 (7–16)	6/25	24 (7–41)	100/820	12 (10–14)
2013	3/195	2 (-0.2–3)	7/163	4 (1–7)	2/140	1 (-0.5–3)	0/17	0 (0–0)	12/515	2 (1–4)
2014	2/271	1 (-0.3–2)	0/179	0 (0–0)	0/81	0 (0–0)	0/0	0 (0–0)	2/531	0.4 (−0.1–1)
2015	18/107	17 (10–24)	11′/79	14 (6–22)	3/52	8 (1–15)	0/4	0 (0–0)	33/242	14 (9–18)
2016	56/224	25 (19–31)	51/138	37 (29–45)	12/81	15 (7–23)	1/2	50 (−19–119)	120/445	27 (23–31)
2017	13/61	21 (11–32)	9′/31	29 (13–45)	8′/34	24 (9–38)	0/3	0 (0–0)	30/129	23 (16–31)
2018	41/544	8 (5–10)	40/402	10 (7–13)	22/240	9 (6–13)	1′/20	5 (−5–15)	104/1206	9 (7–10)
Total	1765/7256	24 (23–25)	976/4819	20 (19–21)	324/2102	15 (14–17)	37/173	21 (15–28)	3102/14350	22 (21–22)
